# Sarcopenia is an effective predictor of difficult-to-wean and mortality among critically ill surgical patients

**DOI:** 10.1371/journal.pone.0220699

**Published:** 2019-08-08

**Authors:** Hao-Wei Kou, Chih-Hua Yeh, Hsin-I Tsai, Chih-Chieh Hsu, Yi-Chung Hsieh, Wei-Ting Chen, Hao-Tsai Cheng, Ming-Chin Yu, Chao-Wei Lee

**Affiliations:** 1 Division of General Surgery, Department of Surgery, Chang Gung Memorial Hospital, Linkou Medical Center, Guishan, Taoyuan, Taiwan, Republic of China; 2 Department of Medical Imaging and Intervention, Chang Gung Memorial Hospital, Linkou Medical Center, Guishan, Taoyuan, Taiwan, Republic of China; 3 College of Medicine, Chang Gung University, Guishan, Taoyuan, Taiwan, Republic of China; 4 Department of Anesthesiology, Chang Gung Memorial Hospital, Linkou Medical Center, Guishan, Taoyuan, Taiwan, Republic of China; 5 Graduate Institute of Clinical Medical Sciences, Chang Gung University, Guishan, Taoyuan, Taiwan, Republic of China; 6 Department of Hepatogastroenterology, Chang Gung Memorial Hospital, Linkou Medical Center, Guishan, Taoyuan, Taiwan, Republic of China; 7 Department of Surgery, Xiamen Chang Gung Hospital, Xiamen, China; Azienda Ospedaliero Universitaria Careggi, ITALY

## Abstract

**Background:**

Critically-ill surgical patients are at higher risk for sarcopenia, which is associated with worse survival. Sarcopenia may impair the respiratory musculature, which can subsequently influence the outcome of ventilator weaning. Although there are a variety of weaning parameters predictive of weaning outcomes, none have tried to incorporate “muscle strength” or “sarcopenia”. The aim of the current study was to explore the association between sarcopenia and difficult-to-wean (DtW) in critically-ill surgical patients. The influence of sarcopenia on ICU mortality was also analyzed.

**Methods:**

Ninety-six patients undergoing mechanical ventilation in the surgical intensive care unit (ICU) were enrolled. Demographic data and weaning parameters were recorded from the prospectively collected database, and the total psoas muscle area (TPA) was determined at the level of the 3^rd^ lumbar vertebra by computed tomography. Sarcopenia was defined by previously established cut-off points and its influence on clinical outcomes was examined. Receiver operating characteristic (ROC) curve analysis was conducted to investigate the predictive capability of TPA and weaning parameters for predicting weaning outcomes.

**Results:**

The median age of the studied patients was 73 years. Thirty patients (31.3%) were sarcopenic and 30 (31.3%) were defined as DtW. Eighteen patients (18.8%) had ICU mortality. Multivariate logistic regression analyses revealed that sarcopenia was an independent risk factor for DtW and ICU mortality. The area under the ROC curve (AUC) of TPA for predicting successful weaning was 0.727 and 0.720 in female and male patients, respectively. After combining TPA and conventional weaning parameters, the AUC for DtW increased from 0.836 to 0.911 and from 0.835 to 0.922 in female and male patients, respectively.

**Conclusion:**

Sarcopenia is an independent risk factor for DtW and ICU mortality. TPA has predictive value when assessing weaning outcomes and can be used as an effective adjunct predictor along with conventional weaning parameters.

## Introduction

In the intensive care unit (ICU), mechanical ventilation should be weaned as early as possible once the clinical condition is stabilized. The decision of whether a patient is ready-to-wean is assessed by various weaning parameters such as the rapid shallow breathing index (RSBI) and several physiological conditions including cardiopulmonary stability [1–4]. Despite favorable weaning profiles, a significant portion of patients, ranging from 10% to 30%, still experience weaning failure or have difficult weaning [5–9]. The pathophysiology of weaning failure is often multifactorial, including comorbidities with dysfunction of the diaphragm/respiratory muscles, lung, and heart [1,2,5]. These patients consume a disproportionate amount of ICU resources [1], are at higher risk of complications such as ventilator-associated pneumonia [10], and more importantly, are associated with increased mortality [6–9,11]. As a result, it is imperative to explore several more effective weaning predictors to improve weaning outcomes.

Sarcopenia is characterized by loss of muscle mass, strength, and function [12,13]. The development of sarcopenia may be related to age, physical activity, nutrition, and diseases such as cancer or chronic inflammation [12,14,15]. Patients with sarcopenia are particularly vulnerable in the presence of major physiologic stressors including major surgery [14] and critical illness [16]. Indeed, in patients who have received major surgery, sarcopenia is associated with poorer outcomes regarding short-term survival, long-term survival, and postsurgical complications [14,17–21]. Likewise, in critically ill patients, sarcopenia is a poor prognostic factor for ventilator-free days, ICU-free days, length of hospital stay, and mortality [15,22–25]. Since sarcopenia may impair the function of respiratory muscles, which in turn influences the weaning process [5,26–29], it is likely that sarcopenia may negatively impact the weaning outcome, especially in surgical patients. However, evidence to support this hypothesis is still lacking. In this retrospective cohort study, we aim to investigate whether sarcopenia is a significant predictive factor for difficult-to-wean (DtW) and ICU mortality in critically ill surgical patients.

## Materials and methods

### Study population and data collection

This study was performed in the 10-bed surgical ICU of a 3500-bed tertiary medical center in Taiwan. From November 2013 to November 2014, all patients who had been intubated and mechanically ventilated during their ICU stay were identified. Patients who had unplanned extubation, who were unable to initiate a spontaneous breathing trial (SBT), who did not have available computed tomography images within 30 days of the first SBT, who had a psoas muscle abscess or received lumbar spine surgery, or whose clinical data were not complete were excluded from our study. A total of 96 patients were eventually enrolled. Their clinical data were retrospectively reviewed from the prospectively collected database. The baseline demographic characteristics, laboratory examinations upon ICU admission, conventional weaning parameters at the first SBT, and total psoas muscle area (TPA) were examined and recorded.

During their ICU stay, weaning was initiated as early as possible. Patients were judged to be ready-to-wean once they fulfilled the criteria, which included 1) a controlled underlying pathology, 2) a stable cardiovascular, metabolic, and mental status, and 3) adequate oxygenation and pulmonary function. The patients regarded as ready-to-wean would undergo a series of SBTs to assess their capability of weaning from the ventilator. The weaning outcome and final survival status were recorded and the patients enrolled were grouped and analyzed accordingly. For internal validation, another 30 patients with identical inclusion/exclusion criteria were randomly selected from the ICU admissions between December 2014 and June 2016.

### Definitions

**Fig 1:**
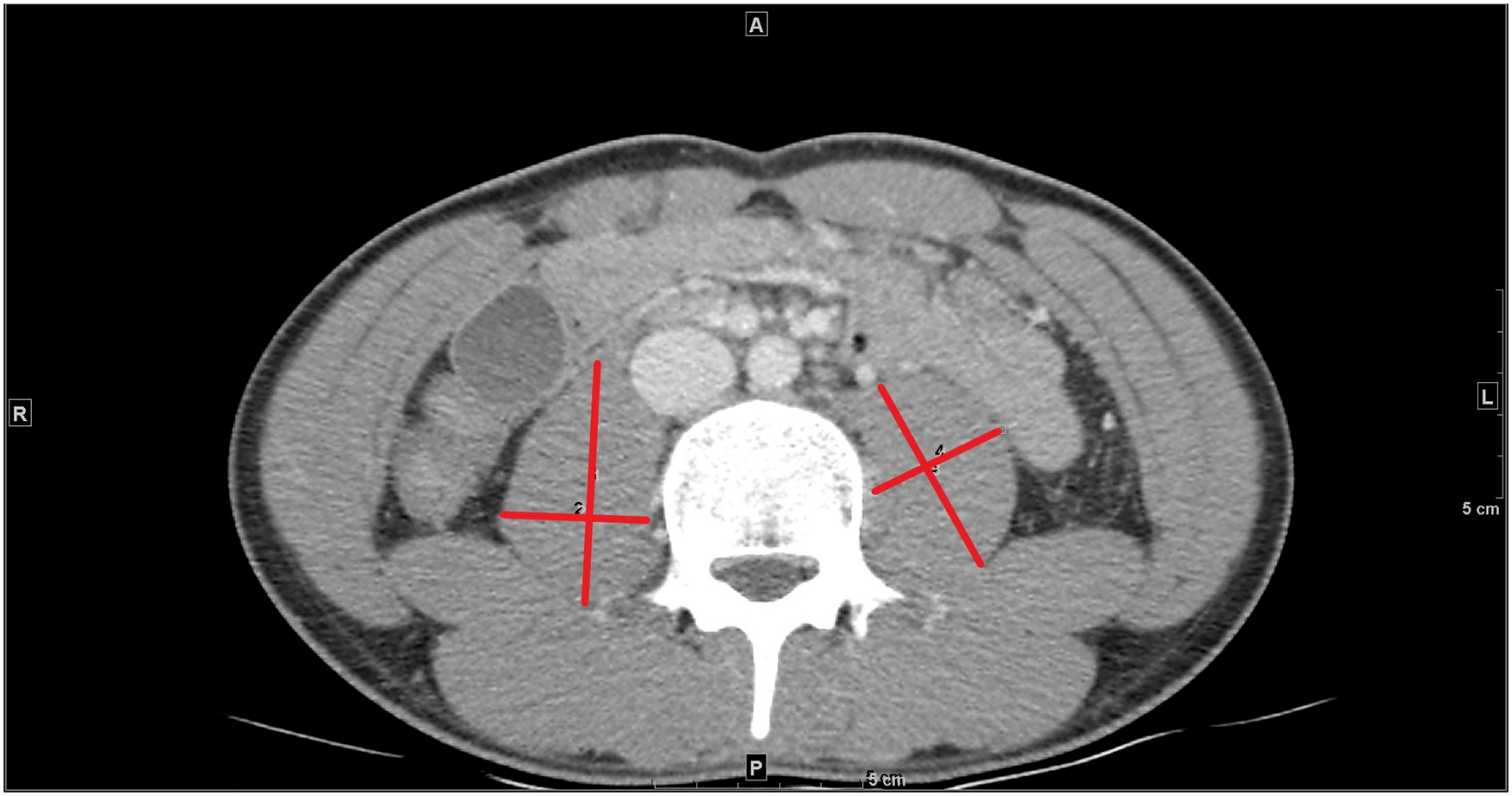
Representative computed tomography image at the level of the L3 vertebral body. The red lines represent the greatest anterior/posterior and transverse diameters of psoas muscle obtained by using a picture archiving and communications system.

The conventional weaning parameters included tidal volume (Vt), respiratory rate (RR), RSBI (determined by RR/Vt), maximal inspiratory pressure (Pimax), and the ratio of the partial pressure of arterial oxygen to the fraction of inspired oxygen (PaO_2_/FiO_2_ ratio). During an SBT, the inspiratory pressure of the pressure support ventilation would be reduced to 8 cmH2O or less. Patients who had RR < 30 breaths/min, Vt > 5 ml/kg, RSBI <105, Pimax >-20 cmH2O, or a PaO_2_/FiO_2_ ratio>200 during their SBT were recognized as potential candidates for ventilator independence. Patients were classified as DtW if they required more than 7 days of weaning after the first SBT (i.e., prolonged weaning) or were reintubated within 48 hours of extubation (i.e., weaning failure) [1,5]. For TPA, a specialized radiologist who was blind to the patients’ clinical condition and outcome estimated the psoas muscle mass. We followed a previously described method that measured the cross-sectional area of the psoas muscle at the level of the third lumbar vertebrae. By using a picture archiving and communication system, the psoas muscle area was estimated by multiplying the greatest anterior/posterior and transverse muscle diameters and then normalizing by the patient’s height [mm^2^/m^2^] (Fig 1) [30, 31]. The values obtained from this method have been shown to correlate well with those obtained from the image-analysis software package [30]. A TPA less than 385 mm^2^/m^2^ for female patients or 545 mm^2^/m^2^ for male patients was defined as sarcopenia [30,31]. Age >75 years was defined as old patient, and operations lasting for more than 5 hours were regarded as prolonged operations.

### Statistical analysis

The Kolmogorov-Smirnov test was used to check the normality of the continuous variables. Continuous variables were analyzed by Student’s t-test for normally distributed data and by the Mann–Whitney U test for non-parametrically distributed data, and are presented as the mean ± standard deviation or median with interquartile ranges for the normally and non-parametrically distributed data, respectively. Pearson’s χ 2 test or Fisher’s exact test was used to compare the categorical variables and was presented as absolute frequency and percentages. Factors that were statistically significant in the univariate analysis were subjected to multivariate logistic regression analysis. The predictive power of individual weaning parameters and various combinations of parameters for weaning outcome was determined by receiver operating characteristic (ROC) curve analysis and was presented as the area under the ROC curve (AUC) [32,33]. P-values less than 0.05 were considered statistically significant. All statistical analyses were performed using IBM SPSS Statistics 21 (IBM Corporation, Software Group, Somers, NY, USA).

## Results

### Patient characteristics

Table 1 demonstrates the demographic data and clinical outcomes of 96 enrolled patients. The median age was 73 years and most (67.8%) were elderly subjects (age ≥ 65 years). Among them, 86 (89.6%) patients received abdominal surgery either immediately before or during their ICU admission, with gastrointestinal surgery being the most common type. More than half of these surgical patients (57.0%) underwent oncological surgery. More than one-third of them (36.0%) received emergent surgery and approximately 30% of all recruited patients suffered from sepsis. Table 2 describes the indications for mechanical ventilation for the patients recruited. The most common indication was moderate/severe restrictive lung disease or elderly patients undergoing emergent abdominal operations, followed by elderly patients with mild restrictive lung disease or sepsis with respiratory failure. The median duration of mechanical ventilation was 5 days and the median duration of mechanical ventilation before being ready-to-wean was 3 days. The mean TPA of the entire patient group was 591.7 mm^2^/m^2^, and 30 patients (31.3%) were classified as having sarcopenia. Thirty patients (31.3%) were classified as DtW, with 25 suffering from prolonged weaning and 5 suffering from weaning failure. Overall, 18 patients (18.8%) died during their ICU stay. Among these patients, 13 were regarded as DtW; DtW was found to be significantly associated with ICU mortality in the current study (ICU mortality, 43.3% in the DtW group *vs*. 7.6% in the non-DtW group, *P*<0.001).

**Table 1:** Demographic characteristics upon ICU admission and clinical outcomes of the study patients.

Variables		
**Age (years)**		73.0 (63.0–80.8)
**Sex**	Male	63 (65.6%)
	Female	33 (34.4%)
**Type of surgery**	Hepatectomy	18 (18.8%)
	Gastrectomy	18 (18.8%)
	Pancreatic surgery	5 (5.2%)
	GI tract surgery	39 (40.6%)
	Urologic surgery	6 (6.3%)
	Without surgery	10 (10.4%)
**Oncological surgery**	Yes	49 (57.0%)
	No	37 (43.0%)
**Emergent surgery**	Yes	31 (36.0%)
	No	55 (64.0%)
**Sepsis**	Yes	28 (29.2%)
	No	68 (70.8%)
**APACHE II score**		16.0 (12.0–20.0)
**SOFA score**		5.0 (3.3–8.0)
**BMI (kg/m**^**2**^**)**		22.8 (20.5–25.2)
**TPA (mm**^**2**^**/m**^**2**^**)**		591.7 ± 212.2
**Male**		639.4 ± 28.1
**Female**		500.6 ± 33.1
**Sarcopenia**	Yes	30 (31.3%)
	No	66 (68.7%)
**ICU morality**	Yes	18 (18.8%)
	No	78 (81.2%)
**ICU stay (days)**		8.5 (4.0–26.8)
**Hospital stay (days)**		32.5 (23–68.5)
**Difficult-to-wean**	Yes	30 (31.3%)
	No	66 (68.7%)
**Duration with MV (days)**		5 (2.0–22.0)
**MV days before readiness to wean (days)**		3.0 (2.0–7.0)

ICU, intensive care unit; GI, gastrointestinal; APACHE, acute physiology and chronic health evaluation; SOFA, sequential organ failure assessment; BMI, body mass index; TPA, total psoas area; MV, mechanical ventilation. Continuous data are expressed as the mean ± standard deviation or median (interquartile range), and categorical data are expressed as a number (%).

**Table 2:** Indications for the use of mechanical ventilation (n = 96).

With prior PFT	n (%)	Without prior PFT	n (%)
**Restrictive lung disease**		**Acute abdomen undergoing emergent operations**	
Severe	4 (4.2)	Shock requiring vasopressors	9(9.4)
Moderate	8(8.3)	Massive transfusion	1(1.0)
Mild, with		Prolonged operative time	3(3.1)
Old age/ comorbidity	10(10.4)	Old age / comorbidity	12(13.0)
Massive transfusion	2(2.1)		
Prolonged operative time	2(2.1)	**Major abdominal operations**	
**Obstructive lung disease**		Shock requiring vasopressors	2(2.1)
Severe	3(3.1)	Massive transfusion	4 (4.2)
Moderate ^a^	5(5.2)	Old age/ comorbidity + prolonged operative time	7(7.3)
Mild, with	2(2.1)		
Old age/ comorbidity		Difficult weaning at OR	1(1.0)
**Normal pulmonary function**			
Old age/ comorbidity with		**Major trauma**	1(1.0)
Massive transfusion	3(3.1)		
Prolonged operative time	6(6.3)	**Sepsis + respiratory failure**	10(10.4)
Difficult weaning at OR	1(1.0)		

PFT, pulmonary function test; OR, operating room

^**a**^Two patients had obstructive lung disease combined with a restrictive pattern

### Predictors of DtW and ICU mortality

Tables 3 and 4 contain the clinical demographic characteristics, laboratory examinations, and weaning parameters with respect to weaning outcome and ICU mortality. As shown in the tables, DtW patients had significantly lower TPA values and significantly higher acute physiology and chronic health evaluation (APACHE) II scores and sequential organ failure assessment (SOFA) scores than those of non-DtW patients. Patients with sarcopenia, sepsis, and those undergoing emergent operations were significantly more likely to be DtW patients. Similar results were also observed between ICU survivors and non-survivors. Furthermore, the conventional weaning parameters RR, Vt, and RSBI were found to be significantly associated with the weaning outcome and are comparable to previous studies [2].

**Table 3:** Clinical demographic characteristics of patients, categorized by weaning outcome and ICU mortality, upon ICU admission.

Variables		Difficult-to-wean			ICU mortality		
		Yes (n = 30)	No (n = 66)	*P* value	Yes (n = 18)	No (n = 78)	*P* value
**Age(years)**		75.5 (63.8–84.5)	72.5 (60.0–80.0)	0.195	67.5 (57.8–77.5)	74.5(63.8–81.0)	0.239
	≥65	22 (33.8)	43 (66.2)	0.427	10 (15.4)	55 (84.6)	0.221
	<65	8 (25.8)	23 (74.2)		8 (25.8)	23 (74.2)	
**Sex**	Male	19 (30.2)	44 (69.8)	0.750	13 (20.6)	50 (79.4)	0.513
	Female	11 (33.3)	22 (66.7)		5 (15.2)	28 (84.8)	
**BMI (kg/m**^**2**^**)**		23.8 (20.5–27.6)	22.3 (20.5–24.5)	0.179	21.8 (20.5–26.8)	23.0 (20.5–25.0)	0.833
**BMI classification**^a^							
	Underweight	5 (41.7)	7 (58.3)	0.068	1 (8.3)	11 (91.7)	0.092
	Normal	8 (21.1)	30 (78.9)		11(28.9)	27 (71.1)	
	Overweight	5 (22.7)	17 (77.3)		1 (4.5)	21 (95.5)	
	Obese	12 (50.0)	12 (50.0)		5 (20.8)	19 (79.2)	
**Emergent operation**^b^	Yes	14 (45.2)	17 (54.8)	**0.007**	9 (29.0)	22 (71.0)	**0.007**
	No	10 (18.2)	45 (81.8)		3 (5.5)	52 (94.5)	
**Sepsis**	Yes	14 (50.0)	14 (50.0)	**0.011**	9 (32.1)	19 (67.9)	**0.031**
	No	16 (23.5)	52 (76.4)		9 (13.2)	59 (86.8)	
**TPA (mm**^**2**^**/m**^**2**^**)**		480.2±188.5	642.3±217.6	**0.001**	474.6±215.3	618.7±215.0	**0.012**
**Sarcopenia**	Yes	14 (46.7)	16 (53.3)	**0.028**	10 (33.3)	20 (66.7)	**0.014**
	No	16 (24.2)	50 (75.8)		8 (12.1)	58 (87.9)	

ICU, intensive care unit; TPA, total psoas area; BMI, body mass index. Continuous data are expressed as the mean ± standard deviation or median (interquartile range) and categorical data are expressed as a number (%). P values <0.05 were considered statistically significant.

^a^ Classification according to the World Health Organization Asia-Pacific criteria

^b^ Ten of the study patients did not have an operation during the study period, but they previously (> 1 year) had surgery in this hospital and were cared for in our ICU, mainly for sepsis.

**Table 4:** Results of the laboratory examination and the weaning profiles of study patients categorized by weaning outcome and ICU mortality.

Variables	Difficult-to-wean			ICU mortality		
	Yes (n = 30)	No (n = 66)	*P* value	Yes (n = 18)	No (n = 78)	*P* value
**Upon admission**						
APACHE II score	20.5 (16.8–24.3)	14.0 (12.0–18.0)	**<0.001**	20.5 (16.0–24.3)	15.0 (12.0–19.0)	**0.001**
SOFA score	7.0 (5.8–10.0)	4.0 (3.0–6.5)	**<0.001**	8.0 (6.0–12.3)	5.0 (3.0–7.0)	**<0.001**
Creatinine (mg/dl)	1.5 (1.0–2.5)	0.8 (0.7–1.3)	**0.003**	1.3 (0.9–2.6)	0.9 (0.7–1.7)	**0.023**
Sodium (mmol/dl)	138 (134–142)	138 (135–140)	0.745	135 (130–141)	138 (135–141)	0.109
Potassium (mmol/dl)	3.9 (3.5–4.6)	4.0 (3.7–4.5)	0.632	3.9 (3.5–4.6)	4.0 (3.6–4.5)	0.728
Albumin (g/dl)	2.3 (2.0–2.7)	3.1 (2.5–3.5)	**<0.001**	2.3 (2.0–3.1)	2.9 (2.4–3.5)	**0.005**
Hemoglobin	10.9±2.0	10.4±1.6	0.157	10.9±2.0	10.4±1.7	0.340
Leukocyte count	11.7 (8.5–20.9)	12.2 (8.7–18.2)	0.953	12.9 (8.7–18.7)	11.9 (8.7–18.8)	0.481
Platelet count	175 (92–245)	182 (129–281)	0.355	169 (72–296)	182 (132–245)	0.551
**Weaning parameters**	**Difficult-to-wean**					
RR < 30 vs. >30 breaths/min	21 (25.9) vs. 9 (60.0)		**0.014**			
Vt >5 vs. <5 ml/kg	14 (21.9) vs. 16 (50.0)		**0.005**			
RSBI <105 vs. >105	19 (25.0) vs. 11 (55.0)		**0.010**			
Pimax <-20 vs. >-20 cmH_2_O	28 (30.1) vs. 2 (66.7)		0.229			
PaO_2_/FiO_2_ ratio>200 vs. <200	28 (29.8) vs. 2 (100.0)		0.095			

ICU, intensive care unit; APACHE, acute physiology and chronic health evaluation; SOFA, sequential organ failure assessment; RR, respiratory rate; Vt, tidal volume; RSBI, rapid shallow breathing index; Pimax, maximum inspiratory pressure; PaO2/FiO2 ratio, the ratio of arterial oxygen partial pressure to fractional inspired oxygen. Continuous data are expressed as the mean ± standard deviation (range) or median (interquartile range) and categorical data are expressed as a number (%). P values <0.05 were considered statistically significant.

Table 5 demonstrates the multivariate logistic regression analysis results for various predictive factors found in the univariate analysis. Sarcopenia (p = 0.038) and APACHE II score (p<0.001) were found to be independent predictors for DtW, whereas RR<30 breaths/min (p = 0.044), RSBI <105 (p = 0.045), and Vt >5 ml/kg (p = 0.004) were shown to significantly decrease the odds of DtW. On the other hand, sarcopenia (p = 0.042), emergent operation (p = 0.033), and SOFA score (p = 0.004) were independent risk factors for ICU mortality.

**Table 5:** Multivariate logistic regression analysis of independent predictive factors for weaning outcome and ICU mortality.

Variables	Difficult-to-wean			ICU mortality		
	OR	95% CI	*P* value	OR	95% CI	*P* value
Sarcopenia	4.767	1.094–20.772	**0.038**	5.071	1.059–24.277	**0.042**
Emergent operation	-	-	NS	5.866	1.152–29.873	**0.033**
Sepsis	-	-	NS	-	-	NS
APACHE II score	1.440	1.201–1.726	**<0.001**	-	-	NS
SOFA score	-	-	NS	1.414	1.116–1.791	**0.004**
Creatinine (mg/dl)	-	-	NS	-	-	NS
Albumin (g/dl)	-	-	NS	-	-	NS
RR<30 breaths/min	0.047	0.002–0.918	**0.044**			
Vt >5 ml/kg	0.063	0.009–0.421	**0.004**			
RSBI <105	0.030	0.001–0.923	**0.045**			

ICU, intensive care unit; APACHE, acute physiology and chronic health evaluation; SOFA, sequential organ failure assessment; RR, respiratory rate; Vt, tidal volume; RSBI, rapid shallow breathing index; OR, odds ratio after adjustment for other confounding factors; CI, confidence interval; NS, no significant difference. P values <0.05 were considered statistically significant.

### ROC curve analyses to predict weaning outcome

**Fig 2:**
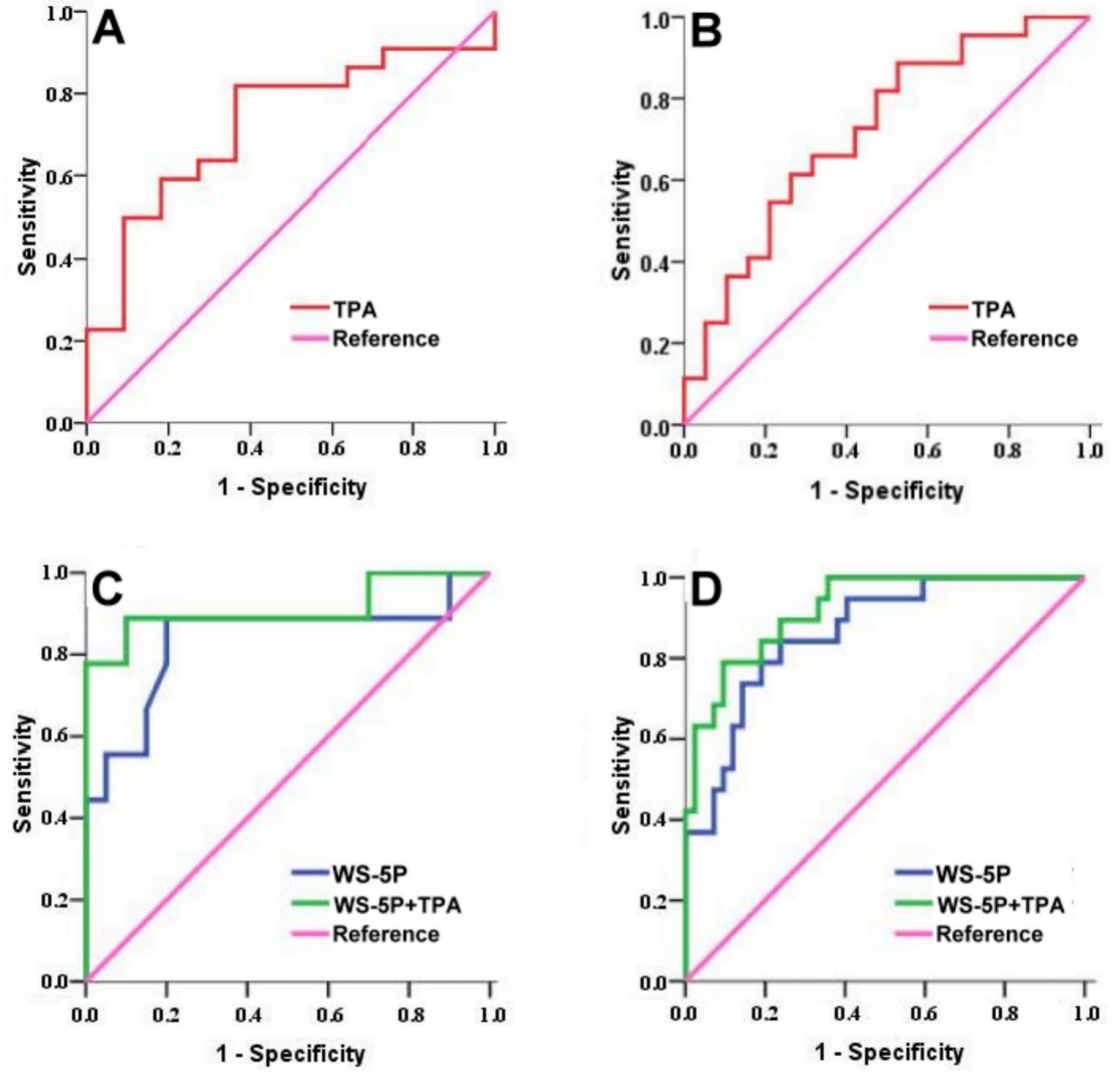
Receiver operating characteristic (ROC) curves for various predictors of weaning outcomes in the study patients. TPA represents the total psoas muscle area. Panels A and B show ROC curves predicting successful weaning when TPA alone was used for analysis of female and male patients, respectively. A logit model of the logistic regression incorporating 5 conventional weaning parameters (WS-5P; with respiratory rate, tidal volume, rapid-shallow breathing index, maximum inspiratory pressure, and PaO_2_/FiO_2_ ratio as the included weaning parameters) or that model in combination with TPA (WS-5P+TPA) was employed to yield a single weaning score. Panels C and D show ROC curves predicting difficult-to-wean (DtW) when WS-5P and WS-5P+TPA were used for analysis of female and male patients, respectively. The purple line is the reference line.

To predict the occurrence of DtW more accurately, ROC curve analysis of various weaning parameters for weaning outcome was employed. The AUC of TPA alone for predicting successful weaning was 0.727±0.091 in female (Fig 2A) and 0.720±0.071 in male patients (Fig 2B) (Table 6). The AUC of 5 individual conventional weaning parameters (Vt, RR, RSBI, Pimax, and PaO_2_/FiO_2_ ratio) for predicting successful weaning or DtW was between 0.688 and 0.752 (Table 6). A logit model of the logistic regression [32,33] incorporating these 5 conventional weaning parameters with and without TPA (WS-5P+TPA and WS-5P, respectrively) was established to yield a single weaning score to predict DtW. The AUC was 0.836±0.097 in female patients and 0.835±0.048 in male patients, when WS-5P was adopted. The addition of TPA into the WS-5P model (i.e., WS-5P+TPA) increased the AUC to 0.911±0.075 and 0.922±0.034 in female and male patients, respectively (Fig 2C and 2D, and Table 6).

**Table 6:** Area under the receiver operating characteristic (ROC) curves for various predictors of weaning outcomes in the study patients.

Predictor	AUC	*P* value	Weaning outcome
TPA (Female)	0.727 ± 0.091	0.036	Success
TPA (Male)	0.720 ± 0.071	0.006	Success
RR	0.700 ± 0.061	0.003	DtW
Vt	0.711 ± 0.058	0.001	Success
RSBI	0.752 ± 0.052	<0.001	DtW
Pimax	0.668 ± 0.063	0.011	DtW
PaO_2_/FiO_2_ ratio	0.732 ± 0.059	<0.001	Success
WS-5P (Female)^a^	0.836 ± 0.097	0.004	DtW
WS-5P (Male)^a^	0.865 ± 0.048	<0.001	DtW
WS-5+TPA (Female)^a^	0.911 ± 0.075	<0.001	DtW
WS-5+TPA (Male)^a^	0.922 ± 0.034	<0.001	DtW

AUC, area under the ROC curve; DtW, difficult-to-wean; TPA, total psoas area; RR, respiratory rate; Vt, tidal volume; RSBI, rapid shallow breathing index; PiMax, maximum inspiratory pressure; PaO2/FiO2 ratio, the ratio of arterial oxygen partial pressure to fractional inspired oxygen. AUC results are presented as the mean ± standard error (SE). *P* values <0.05 were considered statistically significant.

^**a**^ A logit model of the logistic regression incorporating 5 conventional weaning parameters or the same model in combination with TPA (WS-5P or WS-5P+TPA, respectively) was used to determine a single weaning score from the ROC curve analysis.

### Internal validation

Among the 30 patients in the validation group, 21 were male, and 9 were female. Eleven patients (36.7%) were classified as having sarcopenia, and 7 patients (23.3%) were defined as DtW. ROC curve analysis was performed to validate the predictive performance of TPA. The AUC of TPA alone for predicting successful weaning was 0.514 in male and 0.944 in female patients. The addition of TPA into the model with 5 conventional weaning parameters (WS-5P+TPA) increased the AUC for predicting DtW from 0.661 to 0.750 in male patients. The AUCs for the WS-5P and WS-5P+TPA models for predicting DtW were both 1.000 in female patients.

## Discussion

Critically ill surgical patients, especially those with advanced age [21–23] or cancer [18–20], are at higher risk of sarcopenia. Sarcopenia may impair the function of respiratory muscles [26–28], which potentially leads to poor weaning outcomes [5,29]. In the current study, we consecutively enrolled a cohort of critically ill surgical patients from a tertiary medical center in Taiwan. Since more than 80% of our patients underwent abdominal operations, of which nearly 60% were oncological surgery, our patient population was rather homogenous and thus could more accurately allow the investigation of the predictive power of sarcopenia for determining weaning outcomes. The results demonstrated that sarcopenia is a significant predictive factor for DtW among critically ill surgical patients. This finding indicates the importance of skeletal muscle mass or function during ventilator weaning and the necessity of risk stratification prior to the initiation of the weaning process among surgical, especially abdominal surgical, patients.

The current study also noted that among 58 patients whose five conventional weaning parameters were all favorable, 11 patients (19%) were classified as DtW (8 had prolonged weaning, and 3 had weaning failure). This result was comparable to previous studies in which a significant portion of patients are still considered as difficult weaning despite favorable weaning profiles [5–9]. As a result, it is urgently necessary to amend conventional weaning profiles to avoid unnecessary weaning or extubation. After thorough analysis, we discovered from the current study that among these 11 potential weaning candidates, 7 (63.6%) were considered to have sarcopenia. Therefore, we believe that the evaluation of skeletal muscle mass or function should not be neglected and should be incorporated into weaning profiles. Since TPA was found to possess an acceptable discriminative power among successful and non-successful weaners and the weaning outcome could be improved by combining multiple predictors [34], we decided to incorporate TPA into conventional weaning scores to obtain an even better predictive tool. As shown in the results, the AUC for DtW was further enhanced to 0.911 and 0.922 in female and male patients, respectively, when WS-5P+TPA was adopted. Collectively, we believe that TPA is an important variable during ventilator weaning and should be considered along with conventional weaning parameters to optimize the weaning outcome.

In addition to its negative impact on weaning outcomes, sarcopenia was also identified as an independent risk factor for ICU mortality. The incidence of ICU mortality was also significantly higher in the DtW group than in the non-DtW group. It is likely that the same risk factor, i.e., sarcopenia, impairs the weaning process first and then influences ICU survival. Sarcopenia has been reported to be associated with various adverse outcomes in surgical or critically ill patients. For example, a meta-analysis investigating 24 studies and 5,267 patients indicated that radiologically determined sarcopenia predicted morbidity and mortality following abdominal surgery [17]. Another meta-analysis involving 29 studies and 7,176 patients showed that sarcopenia was associated with an increased risk of postoperative complications in patients with gastrointestinal cancer [19]. Other recent studies also reported that sarcopenia was an independent prognostic factor for complications and survival in either surgical or nonsurgical patients [20–22]. A decrease in skeletal muscle mass as defined by cross-sectional CT imaging has been demonstrated to be a risk factor for in-hospital mortality in ICU patients of old or nonspecific age [23–25]. With the aforementioned evidence, we conclude that TPA or sarcopenia is a rather important parameter in surgical ICUs and should routinely be assessed in critically ill surgical patients.

Since patients in surgical ICUs are usually immobilized or disabled, accurate assessment of their muscle strength is not feasible and a more subjective modality is warranted. It has been suggested that sarcopenia can be assessed by CT imaging techniques in patients in whom measurements of muscle mass, muscle strength, and physical activity are not available [12]. In the current study, we employed CT imaging and a predetermined sex-specific TPA cut-off to estimate TPA and define sarcopenia. The incidence of sarcopenia was comparable to previous studies investigating cancer patients undergoing abdominal surgery (15–50%) and elderly patients suffering from trauma or surgery (24.9–57.5%) [17,21,30,31,35,36]. Since cross-sectional CT imaging is commonly performed in patients scheduled to receive abdominal operations, measurements of TPA are readily available and will not introduce further radiation exposure in this patient subgroup. Therefore, this simple CT imaging technique is a practical and reliable method to assess skeletal muscle mass and sarcopenia in critically ill surgical patients. We do not necessarily require other dedicated computer software, massive calculations, or state-of-the-art instruments to diagnose sarcopenia in this special subgroup of patients.

Despite the remarkable findings, our study still has several limitations. First, the retrospective nature of the current study renders selection bias and missing data inevitable. Second, the size of the validation group is rather small, resulting in less significant results. Third, the current study lacks external validation to support our findings. Future prospective investigations with a larger sample size and external validation group are warranted to validate our findings.

## Conclusions

In conclusion, our study demonstrates that sarcopenia is an independent risk factor for DtW and ICU mortality in critically ill surgical patients. TPA has prognostic significance in predicting weaning outcomes and should be considered along with other conventional weaning parameters in surgical ICUs. Further studies are necessary to confirm our findings.

## Supporting information

S1 Dataset Minimal anonymized dataset necessary to replicate the study finding. (XLS)

## Abbreviations

APACHE: Acute physiology and chronic health evaluation
AUC: Area under the receiver operating characteristic curve
CGMH: Chang Gung Memorial Hospital
CT: computed tomography
DtW: Difficult-to-wean
ICU: Intensive care unit
OR: Operating room
PaO_2_/FiO_2_ ratio: The ratio of partial pressure of arterial oxygen to the fraction of inspired oxygen
PFT: Pulmonary function test
Pimax: Maximal inspiratory pressure
ROC curve: Receiver operating characteristic curve
RR: Respiratory rate
RSBI: Rapid shallow breathing index
SBT: Spontaneous breathing trial
SOFA: Sequential organ failure assessment
TPA: Total psoas muscle area
Vt: Tidal volume
WS-5P +TPA: Weaning score-5 conventional weaning parameters + total psoas muscle area
WS-5P: Weaning score-5 conventional weaning parameters
